# Leukocyte concentrations of inpatients with anorexia nervosa

**DOI:** 10.3389/fpsyt.2026.1812727

**Published:** 2026-04-29

**Authors:** Zuo Zhang, Can Xu, Risha Govind, Johanna Louise Keeler, Robert Stewart, Janet Treasure, Hubertus Himmerich

**Affiliations:** 1 Institute for Mental Health, School of Psychology, University of Birmingham, Birmingham, United Kingdom; 2 Social Genetic and Developmental Psychiatry Centre, Institute of Psychiatry, Psychology, and Neuroscience, King’s College London, London, United Kingdom; 3 Centre for Research in Eating and Weight Disorders (CREW), Department of Psychological Medicine, Institute of Psychiatry, Psychology, and Neuroscience, King’s College London, London, United Kingdom; 4 South London and Maudsley National Health Service (NHS) Foundation Trust, London, United Kingdom; 5 Department of Psychological Medicine, Institute of Psychiatry, Psychology and Neuroscience, King’s College London, London, United Kingdom

**Keywords:** anorexia nervosa, eating disorders, electronic health record, health of the nation outcome scales, leukocytes

## Abstract

**Introduction:**

The leukocyte or white blood cell (WBC) count is a crucial clinical risk marker for anorexia nervosa (AN). Leukocytes can be further classified as granulocytes (e.g., neutrophils, eosinophils) and agranulocytes (e.g., lymphocytes, monocytes). The immature granulocyte count (IGC) has been reported to be associated with psychological stress. However, the IGC has not been investigated in people with AN yet.

**Methods:**

We retrieved complete datasets from the Clinical Records Interactive Search (CRIS) register for 267 inpatients with AN and available leukocyte parameters (total WBC, neutrophils, eosinophils, lymphocytes, monocytes), Health of the Nation Outcome Scales (HoNOS) scores and body mass index (BMI) data at admission. In 49 of these patients, the immature granulocyte count was also available.

**Result:**

Leukocyte baseline levels were WBC: 4.82 × 10^9^/L ± 2.16 (standard deviation), neutrophils: 2.96 × 10^9^/L ± 1.78, eosinophils: 0.08 × 10^9^/L ± 0.1, lymphocytes: 1.46 × 10^9^/L ± 0.57, monocytes: 0.29 × 10^9^/L ± 0.14, IGC: 0.02 × 10^9^/L ± 0.07. The WBC, eosinophils, and monocytes concentration increased significantly from admission to discharge (Cohen’s d = 0.27, 0.32, and 0.65, respectively, *p*s < 0.001). Patients showed a significant increase in BMI from 14.13 kg/m^2^ ± 1.48 to 16.22 kg/m^2^ ± 2.67 (Cohen’s d = 0.77, *p* < 0.001). The HoNOS total score was not significantly associated with any of the leukocyte parameters.

**Discussion:**

In inpatients with AN, the WBC, eosinophil and monocyte count seem to increase alongside weight restoration. Leukocytes did not exhibit reliable associations with health and social functioning measures in this sample.

## 1 Introduction

Anorexia nervosa (AN) is a complex and severe psychiatric disorder characterised by self-induced weight loss, an intense fear of gaining weight, and significant body image distortion (1, 2). Individuals struggling with AN often experience intense emotional and psychological distress, which can severely impact their relationships, and overall quality of life (1, 3). Understanding the causes, symptoms, and treatment options for AN is crucial in supporting those affected.

Studies have investigated changes in serum concentrations of specific inflammatory markers in patients with eating disorders, including pro-inflammatory cytokines like interleukin (IL)-6, C-reactive protein (CRP), and leukocytes (4–7). AN is often associated with a notably low peripheral leukocyte count, also known as low white blood cell count (WBC) or leukocytopenia. Leukocytopenia, a condition indicating bone marrow suppression due to self-induced starvation, may increase the risk of severe infections, including pneumonia, mitral endocarditis, and septicaemia (8, 9). A recent systematic review and meta-analysis based on 38 studies reported the comparison of concentration of leukocytes and different subtypes of leukocytes (basophils, eosinophils, lymphocytes, monocytes, neutrophils) between EDs and healthy controls (10). Peripheral concentrations of leukocytes, monocytes, neutrophils, basophils and lymphocytes were found to be reduced in AN compared to controls.

Leukocytes can be broadly classified into granulocytes—such as neutrophils and eosinophils, and agranulocytes—such as lymphocytes and monocytes (11). Recent research has drawn attention to the immature granulocyte count (IGC), a parameter that reflects the presence of early-stage granulocytes in the bloodstream. IGC concentrations increase in response to physical or psychological stress, inflammation, and various immune-related diseases (12–18). Given that AN is associated with autoimmune and inflammatory disorders (19), as well as shifts in immunological laboratory parameters such as altered cytokine levels (10), C-reactive protein (7), leukocyte concentrations (10), and bone marrow morphology (20), we investigated whether IGC levels are similarly linked to the disorder. To date, however, no studies have evaluated IGC levels in individuals with AN.

We utilised the Clinical Record Interactive Search (CRIS) database to extract data on the leukocytes and subtypes of leukocyte levels, and co-morbid clinical diagnoses. The CRIS database, developed by the South London and Maudsley National Health Service (NHS) Foundation Trust (SLaM) (21), provides anonymised data from the electronic clinical records system. It includes routinely collected information, such as the clinical examination parameters like BMI, and laboratory values such as leukocyte levels.

For the measurement of clinical and psychosocial problems on admission and discharge, we used the Health of the Nation Outcome Scales (HoNOS). The HoNOS provides information on agitated behaviour, self-injury, problem drinking and drug use, cognitive problems, physical illness, hallucinations, depressed mood, relationship problems, daily living problems, problems with living conditions and occupational problems and is the most widely used clinical outcome tool by psychiatric services in England (22, 23). So far, no study has explored a potential association between leukocyte parameters and the HoNOS score, or reported the IGC levels in AN. Previous studies on cytokine parameters in hospitalised inpatients were comparably small with less than 60 study participants.

This study investigates:

- The longitudinal changes in total WBC and leukocyte subtype concentrations (eosinophils, lymphocytes, monocytes, neutrophils, IGC) in inpatients with AN from admission to discharge.
- Associations of WBC and leukocyte subtypes with BMI.
- Associations of WBC and leukocyte subtypes with psychosocial functioning as measured by the HoNOS.

## 2 Methods

### 2.1 Data source

Anonymous patient data were extracted from the Clinical Record Interactive Search (CRIS) database, hosted at the South London and Maudsley NHS Foundation Trust (SLaM). The CRIS source data project was approved by the Oxfordshire Research Ethics Committee C (reference 23/SC/0257). This specific project was approved by the CRIS Oversight Committee (reference 23-044; approval date: 15th of June 2023).

### 2.2 Participants

Patients included in this study were those admitted to the inpatient ward of the Eating Disorders Unit at SLaM from 2006 to 2023. The selected patients were above 18 years of age at admission, with discharge dates available, and had a diagnosis of an eating disorder coded as F50, or a specific diagnosis of AN coded as F50.0, according to the International Statistical Classification of Diseases and Related Health Problems 10th Revision (ICD-10). Information on eating disorder diagnoses was sourced from the CRIS database. Diagnoses were established through routine clinical assessment by a psychiatrist, integrating the patient’s medical history and physical exam with input from family members and local health services. Notably, the diagnoses relied on clinical judgment rather than standardised tools.

We further selected underweight patients for analysis, who had a body mass index (BMI) lower than 18.5 kg/m^2^ at admission.

This approach might have led to some diagnostic heterogeneity. However, in the clinical database that we used, patients with AN were either coded with F50.0 or with F50 by the clinicians. In order not to lose people with AN coded as F50, we used a maximum BMI of 18.5 kg/m^2^ at admission as second criterion. We may have involuntarily included patients with avoidant restrictive food intake disorder (ARFID). However, this diagnosis was only introduced into the DSM-5 in 2013 (1), and this study covers inpatient admissions from 2006 to 2023. Additionally, CRIS has not adopted DSM-5 for diagnostic coding yet but still uses ICD-10 (2).

### 2.3 Measures

The leukocytes measures included eosinophils, lymphocytes, monocytes, neutrophils, WBC, and IGC. The neutrophils to lymphocytes ratio (NLR) was calculated. These were taken from the measurements closest to, and within two weeks of the admission and discharge dates. Nurses completed the HoNOS, a 12-item instrument used to assess patients’ clinical problems and psychosocial functioning at admission and discharge. Each item is rated on a five-point Likert scale. The HoNOS total score was calculated by summing the scores of all 12 items. BMI was measured within 2 weeks of admission and discharge, respectively. The ethnicity variable, originally comprising 14 subcategories, was recoded into a binary variable indicating whether or not a patient identified as White, due to the predominance of White ethnicity in the sample. We also captured whether patients had a diagnosis of depressive disorders (ICD-10 F30, F31, or F32), anxiety disorders (F40, or F41), or personality disorders (F60-69), before or on the admission date. As with the eating disorder diagnoses, comorbid conditions were established through routine clinical assessment and psychiatric judgment, rather than via standardised diagnostic instruments.

Smoking status, the use of antidepressants and antipsychotics were coded as binary variables indicating whether these occurred within one year before admission.

### 2.4 Statistical analysis

We investigated the changes of HoNOS scores, BMI, and leukocyte levels between admission and discharge, by using paired t-tests. To account for confounding variables, we repeated the analyses using linear mixed models. The time of assessment (admission/discharge) was modelled as a fixed effect and binary variable. The effect of patient was modelled as a random variable, forming a random intercept model. Control variables included age, ethnicity, BMI at admission, duration of admission, smoking status, antidepressant and antipsychotic use, and psychiatric comorbidities (depressive disorders, anxiety disorders, and personality disorder). The linear mixed models were fitted using the lme4 package v2.0-1 (24) under R Statistical Software v4.5.3 (25).

Next, we used linear regression to assess the associations between leukocyte levels and sample characteristics (age, BMI and HoNOS total scores) at admission. The association with age did not involve any control variables. The associations with BMI and HoNOS total scores were adjusted for the same control variables as those in the linear mixed models described above. For each analysis, patients with missing values on any required variable were excluded. The p-value threshold was corrected with the Bonferroni correction.

## 3 Results

### 3.1 Sample characteristics

**Figure 1:**
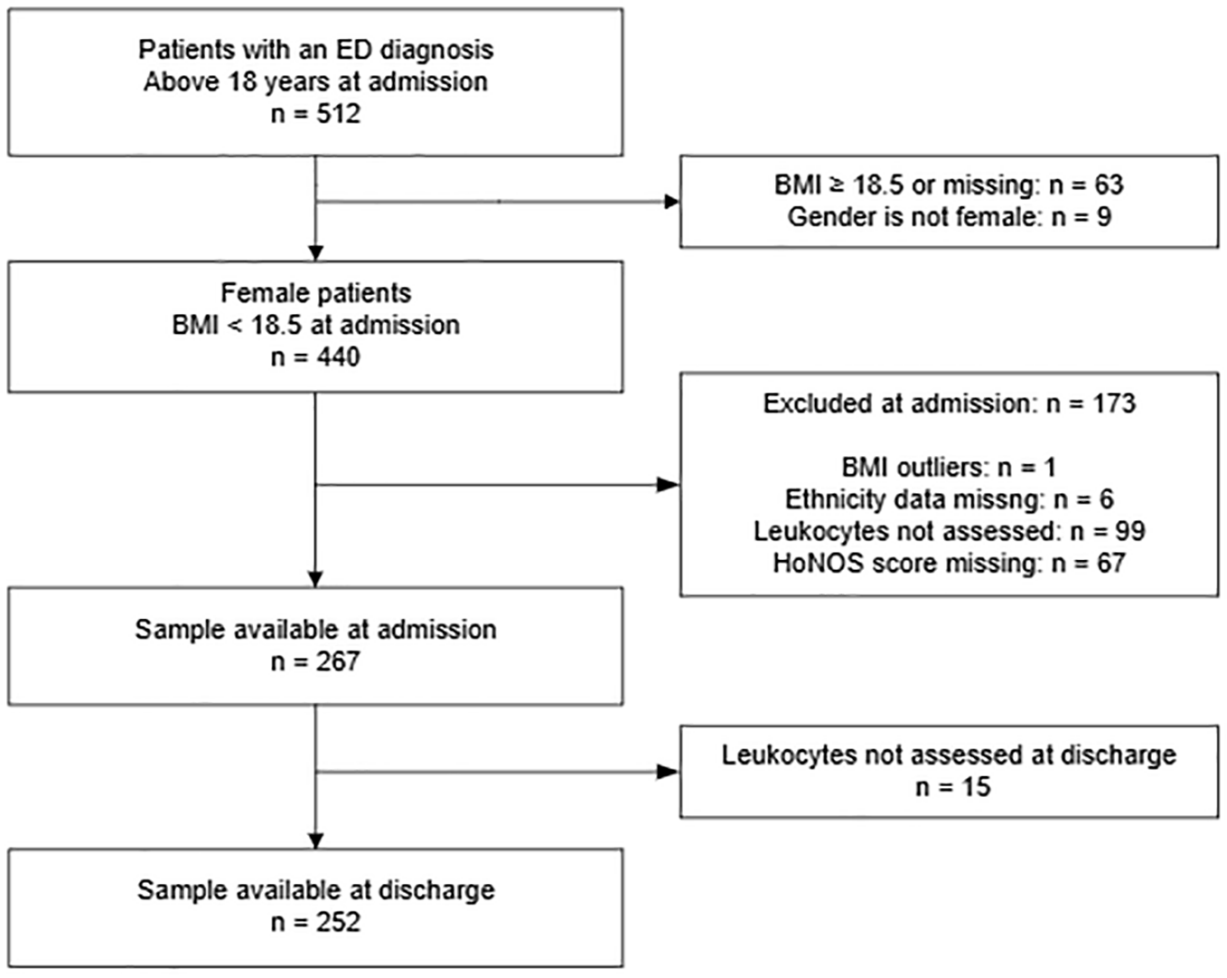
Flow-chart for patient selection.

A total of 512 patients with an ED diagnosis and above 18 years at admission were identified from the CRIS database. We selected female patients who had a BMI lower than 18.5 kg/m^2^ at admission, and had the following variables available: age, ethnicity, HoNOS scores, and at least one of the leukocyte measures (eosinophils, lymphocytes, monocytes, neutrophils, WBC, or IGC). One patient was excluded due to an outlier BMI value that was likely incorrect (BMI = 4.7 kg/m^2^). A final sample of 267 female patients were analysed at point of admission (**Figure 1**, **Table 1**).

**Table 1:** Summary of sample characteristics and changes in HoNOS, BMI and leukocytes from admission to discharge.

Timepoint	Admission				Discharge				Discharge vs. admission	
Variable	N	Mean (SD)/N^+^ (%)	min	max	N	Mean (SD)/N^+^ (%)	min	max	Cohen’s d	P
Age (years)	267	29.26 (11.70)	18	70	–	–	–	–	–	–
Duration from Admission to Discharge (days)	267	114.99 (91.75)	0	618	–	–	–	–	–	–
Ethnicity (white or not) ^+^	267	234 (88%)	–	–	–	–	–	–	–	–
Depressive Disorders ^+^	267	29 (11%)	–	–	–	–	–	–	–	–
Anxiety Disorders ^+^	267	26 (10%)	–	–	–	–	–	–	–	–
Personality Disorder ^+^	267	34 (13%)	–	–	–	–	–	–	–	–
Antidepressants Use ^+^	267	135 (51%)	–	–	–	–	–	–	–	–
Antipsychotics Use ^+^	267	73 (27%)	–	–	–	–	–	–	–	–
Smoking Status ^+^	267	91 (34%)	–	–	–	–	–	–	–	–
Total HoNOS Score	267	14.2 (5.95)	4	44	162	10.04 (4.65)	1	25	-0.63	1.7E-13*
BMI (kg/m^2^)	267	14.13 (1.48)	10.20	18.4	218	16.22 (2.67)	12	30	0.77	8.8E-24*
Eosinophils (10^9^/L)	267	0.080 (0.10)	0	0.84	252	0.16 (0.26)	0	3.31	0.32	6.6E-07*
Lymphocytes (10^9^/L)	267	1.46 (0.57)	0.37	3.93	252	1.52 (0.54)	0.38	4.3	0.16	0.013
Monocytes (10^9^/L)	267	0.29 (0.14)	0.09	0.97	252	0.38 (0.17)	0.12	1.16	0.65	3.8E-21*
Neutrophils (10^9^/L)	267	2.96 (1.78)	0.63	16.19	252	3.19 (1.37)	0.83	9.57	0.14	0.023
WBC (10^9^/L)	267	4.82 (2.16)	1.59	18.07	252	5.29 (1.73)	1.43	11.29	0.27	2.6E-05*
IGC (10^9^/L)	49	0.020 (0.07)	0	0.52	56	0.010 (0.020)	0	0.12	-0.090	0.54
NLR	267	2.19 (1.34)	0.56	11.65	252	2.26 (1.13)	0.44	7.85	0.036	0.57

Cross signs (+) indicate binary variables. N^+^ indicates number of positive cases (those coded as 1) for a binary variable. The comparison between discharge and admission is conducted using paired *t*-tests. Asterisks (*) indicate p values that survive the Bonferroni correction for the 9 tests (adjusted threshold α = 0.0056). NLR, neutrophils to lymphocytes ratio.

This sample had a mean age of 29.26 years (ranging 18-70) and a mean BMI of 14.13 kg/m^2^ (SD = 1.48). IGC measures were available only for a limited number of patients (N = 49). A substantial proportion of patients demonstrated leukocyte counts below the normal range, particularly for eosinophils, with 77.5% of patients showing below-normal values at admission and 49.2% at discharge. On the contrary, only a small number of patients exhibited leukocyte counts above the normal range. **Table 2** depicts the number of participants whose leukocyte levels fall outside the normal range on admission and discharge.

**Table 2:** Number of participants whose leukocyte levels fell outside the normal range.

		Admission			Discharge		
Measure	Normal range	Sample size	N (%) below the normal range	N (%) above the normal range	Sample size	N (%) below the normal range	N (%) above the normal range
Eosinophils (10^9^/L)	0.1-0.4	267	207 (77.5%)	7 (2.6%)	252	124 (49.2%)	14 (5.6%)
Lymphocytes (10^9^/L)	1.1-4.0	267	73 (27.3%)	0 (0%)	252	42 (16.7%)	1 (0.4%)
Monocytes (10^9^/L)	0.2-1.1	267	62 (23.2%)	0 (0%)	252	18 (7.1%)	1 (0.4%)
Neutrophils (10^9^/L)	1.5-8.0	267	27 (13.9%)	4 (1.5%)	252	18 (7.1%)	1 (0.4%)
WBC (10^9^/L)	4.0-11.0	267	102 (38.2%)	5 (1.9%)	252	61 (24.2%)	1 (0.4%)
IGC (10^9^/L)	–	49	–	–	56	–	–

The normal range of leukocyte levels are obtained from the SLaM clinical system. The normal range for IGC is not available.

### 3.2 Change of leukocyte levels from admission to discharge

At discharge, the patients showed significant increases in BMI (Cohen’s d = 0.77, t = 11.36, p = 8.8E-24) and decreases in HoNOS total scores (d = -0.63, t = -8.06, p = 1.7E-13) compared with admission using paired t-tests. Significant increases in eosinophils (d = 0.32, t = 5.10, p = 6.6E-07), monocytes (d = 0.65, t = 10.35, p = 3.8E-21), and WBC levels (d = 0.27, t = 4.28, p = 2.6E-05) were also observed (**Table 1**). These results remained significant when we repeated the analyses using linear mixed models, controlling for the effects of age, ethnicity, BMI at admission, duration of admission, smoking status, antidepressant and antipsychotic use, and psychiatric comorbidities (depressive disorders, anxiety disorders, and personality disorder) (**Table 3**).

**Table 3:** Results of mixed linear models for comparisons of HoNOS, BMI and leukocyte levels at discharge vs. admission.

Variable	Coefficient	SE	t	df	P	Sample size
HoNOS total score	-4.16	0.49	-8.44	185.26	9.1E-15*	162
BMI	2.08	0.16	12.79	473.00	2.2E-32*	218
Eosinophils	0.078	0.016	5.05	261.67	8.3E-07*	252
Lymphocytes	0.072	0.031	2.32	254.57	0.021	252
Monocytes	0.093	0.0090	10.38	258.15	2.5E-21*	252
Neutrophils	0.22	0.10	2.23	257.44	0.026	252
WBC	0.47	0.11	4.19	256.73	3.9E-05*	252
IGC	-0.0076	0.0093	-0.82	93.00	0.41	48
NLR	0.060	0.089	0.68	258.18	0.50	252

Controls variables included age, BMI at admission, duration from admission to discharge, smoking status, antidepressant and antipsychotic use, and psychiatric comorbidities (depressive disorders, anxiety disorders, and personality disorder). Asterisks (*) indicate p values that survive the Bonferroni correction for the 9 tests (adjusted threshold α = 0.0056). HoNOS, Health of the Nation Outcome Scales; SE, standard error; df, degree of freedom.

### 3.3 Associations between leukocyte levels and sample characteristics at admission

We assessed the associations between leukocyte levels and sample characteristics at admission, including age, BMI and HoNOS total scores (**Table 4**). The eosinophils, lymphocytes, and WBC levels showed nominally significant associations with BMI (ps < 0.05), but these associations did not survive the Bonferroni correction. The monocytes, neutrophils, NLR, and WBC levels showed significant positive associations with age (ps < 0.001), surviving the Bonferroni correction. The associations between leukocyte levels and HoNOS total scores were not significant (ps > 0.2).

**Table 4:** Associations of leukocytes with age, BMI, and HoNOS total scores at admission.

Blood cells	Independent variable	Standardised beta	SE	t value	*p*-Value	Sample size
Eosinophils	Age	0.072	0.061	1.17	0.24	267
	BMI	0.174	0.061	2.87	0.0045	267
	HoNOS total	-0.074	0.062	-1.18	0.24	267
Lymphocytes	Age	-0.062	0.061	-1.01	0.31	267
	BMI	0.164	0.060	2.72	0.0070	267
	HoNOS total	0.067	0.062	1.09	0.27743	267
Monocytes	Age	0.29	0.059	5.01	5.3E-05*	267
	BMI	0.108	0.063	1.71	0.088	267
	HoNOS total	-0.012	0.064	-0.18	0.85	267
Neutrophils	Age	0.24	0.060	4.01	7.7E-05*	267
	BMI	0.102	0.062	1.65	0.10	267
	HoNOS total	0.040	0.063	0.64	0.52	267
WBC	Age	0.20	0.060	3.40	7.7E-04*	267
	BMI	0.143	0.061	2.34	0.020	267
	HoNOS total	0.046	0.063	0.74	0.46	267
IGC	Age	-0.0025	0.11	-0.022	0.98	49
	BMI	0.048	0.20	0.24	0.81	49
	HoNOS total	0.043	0.20	0.21	0.83	49
NLR	Age	0.32	0.058	5.53	7.7E-08*	267
	BMI	-0.016	0.064	-0.25	0.80	267
	HoNOS total	0.012	0.065	0.19	0.85	267

Asterisks (*) indicate *p* values that survive the Bonferroni correction for the 21 tests (adjusted threshold α = 0.0024). The association with age did not control for any confounding variables. The associations with BMI and HoNOS total scores were adjusted for age, ethnicity, BMI at admission, duration of admission, smoking status, antidepressant and antipsychotic use, and psychiatric comorbidities (depressive disorders, anxiety disorders, and personality disorder). Bold values indicate statistical significance.

## 4 Discussion

This study examined leukocyte parameters and their changes in underweight inpatients diagnosed with AN using routinely collected clinical data. This is to our knowledge the biggest study in inpatients with AN that measures leukocyte parameters. Previous comparable studies in inpatients included up to 60 inpatients, for example Funayama et al. (26). From admission to discharge, patients showed a significant increase in the WBC, monocyte and eosinophil concentration, and a decrease in HoNOS total score. During their inpatient admission, the mean BMI increased significantly.

Our findings are consistent with previous literature reporting that AN is commonly associated with leukopenia and suppression of immune function due to malnutrition (5, 8). The observed increases in WBC and eosinophil levels during weight restoration likely reflect a normalisation of haematological parameters as nutritional status improves. These improvements may suggest partial reversal of bone marrow suppression, which is common in severe starvation.

The eosinophil, lymphocyte and WBC levels showed positive associations with BMI at admission, which were nominally significant but did not survive the Bonferroni correction. This trend is in line with previous work that linked low WBC in AN to undernutrition (27).

The immature granulocyte count (IGC) did not show significant variation between admission and discharge, nor did it associate with BMI, age, or HoNOS scores. This finding is notable, as prior studies outside of the eating disorder field have reported associations between IGC and psychological stress or inflammatory responses (28). However, the limited sample size for IGC (N = 49) may have restricted our ability to detect subtle effects, and further research in larger samples is needed to clarify this relationship.

Notably, no leukocyte parameter, including IGC, was significantly associated with HoNOS scores. This suggests that while leukocyte counts may serve as useful markers of physical health improvement alongside weight restoration in AN, they are unlikely to capture broader aspects of mental health or social functioning. The lack of association between leukocyte parameters and psychosocial functioning as measured by the HoNOS suggests that leukocyte markers may primarily reflect physiological rather than psychological improvement.

This study has several strengths, including the use of a large, naturalistic clinical sample and the examination of both traditional and novel leukocyte subtypes across admission and discharge.

However, our study has several limitations. The main limitation is that we did not have an age-matched control group of healthy female patients. The patients involved in the analysis were female only and were mostly of white ethnicity. It remains to be tested whether the findings are generalisable to male patients and other ethnicities. As physical health diagnoses including inflammatory and infectious diseases were not consistently and systematically coded in the clinical record system of the psychiatric hospital, we were unable to test reliably whether such conditions have influenced our results. Similarly, medications (e.g., antidepressants and antipsychotics) which were used by a substantial proportion of patients may have affected leukocyte parameters.

Leukocyte measurements were obtained within two weeks of admission and discharge. Therefore, the exact timing of the blood tests might have varied across patients. Usually, inpatients have taken their blood on a weekly basis. For various reasons, however, there might not be a blood test available in the CRIS database on the exact date or within the exact week of admission for various reasons. A patient might have refused to have their blood taken in the week of admission, it might have been impossible to take bloods due to the fragile veins of a person with AN, blood might have been taken in a different hospital that did not feed the blood results into CRIS or blood might have been taken, but it might have been impossible to analyse the blood or it might have been impossible to document the results due to transport, laboratory or computer problems. As leukocyte parameters do not fluctuate rapidly and usually take time to recover after starvation, we decided that a two-week window would be appropriate. Nonetheless, we cannot exclude that the variability of the time point of blood collection could have influenced the results.

The coding of ICD-10 diagnoses was not consistently specific across all cases. As a result, we included patients with a generic eating disorder diagnosis (F50) if they presented with significantly low body weight. It is possible that some of these individuals may have met criteria for avoidant/restrictive food intake disorder (ARFID). However, ARFID was only formally introduced in the DSM-5 in 2013 [1], and our sample includes inpatients treated between 2006 and 2023. During much of this period, the adult inpatient eating disorders service at SLaM primarily catered to individuals with AN, and tailored interventions for ARFID were only implemented more recently. Therefore, it is unlikely that a substantial number of ARFID patients were included in this sample.

The IGC which has been previously linked to psychological stress did not change significantly during treatment and showed no association with clinical or functional measures. However, IGC data were only available for a subset of the sample, reducing statistical power in this analysis. Therefore, these results should be interpreted with caution.

Overall, we would like to point out that the study design was observational and based on routinely collected clinical data; therefore, the study results cannot establish a causal relationship between weight restoration and the reported leukocyte changes.

## Data Availability

Data are owned by a third party, Maudsley Biomedical Research Centre (BRC) Clinical Records Interactive Search (CRIS) tool, which provides access to anonymised data derived from SLaM electronic medical records. These data can only be accessed by permitted individuals from within a secure firewall (i.e. the data cannot be sent elsewhere), in the same manner as the authors. For more information please contact: cris.administrator@slam.nhs.uk. Requests to access the datasets should be directed to cris.administrator@slam.nhs.uk.

## References

[1] American Psychiatric Association . Diagnostic and statistical manual of mental disorders (5th ed.). (2013). doi: 10.1176/appi.books.9780890425596, PMID:

[2] World Health Organization . nternational Statistical Classification of Diseases and Related Health Problems. 10th ed (2016). Available online at: https://www.who.int/browse10/2016/en (Accessed February 10, 2026).

[3] Arcelus J Mitchell AJ Wales J Nielsen S . Mortality rates in patients with anorexia nervosa and other eating disorders. Arch Gen Psychiatry. (2011) 68:724. doi: 10.1001/archgenpsychiatry.2011.74. PMID: 21727255

[4] Butler MJ Perrini AA Eckel LA . The role of the gut microbiome, immunity, and neuroinflammation in the pathophysiology of eating disorders. Nutrients. (2021) 13:500. doi: 10.3390/nu13020500. PMID: 33546416 PMC7913528

[5] Solmi M Veronese N Favaro A Santonastaso P Manzato E Sergi G . Inflammatory cytokines and anorexia nervosa: A meta-analysis of cross-sectional and longitudinal studies. Psychoneuroendocrinology. (2015) 51:237–52. doi: 10.1016/j.psyneuen.2014.09.031. PMID: 25462897

[6] Walsh K Blalock DV Mehler PS . Hematologic findings in a large sample of patients with anorexia nervosa and bulimia nervosa. Am J Hematol. (2020) 95:98–101. doi: 10.1002/ajh.25732. PMID: 31944357

[7] Xu C Mutwalli H Haslam R Keeler JL Treasure J Himmerich H . C-reactive protein (CRP) levels in people with eating disorders: A systematic review and meta-analysis. J Psychiatr Res. (2025) 181:653–62. doi: 10.1016/j.jpsychires.2024.12.039. PMID: 39742796

[8] Himmerich H Schönknecht P Heitmann S Sheldrick AJ . Laboratory parameters and appetite regulators in patients with anorexia nervosa. J Psychiatr Pract. (2010) 16:82–92. doi: 10.1097/01.pra.0000369969.87779.1c. PMID: 20511732

[9] Himmerich H Keeler JL King JA Ehrlich S Kaufmann L-K Bulik CM . World Federation of Societies of Biological Psychiatry (WFSBP) consensus statement on candidate biomarkers for anorexia nervosa. World J Biol Psychiatry. (2026) 27:257–348. doi: 10.1080/15622975.2026.2626934. PMID: 41765047

[10] Keeler JL Bovenberg C Himmerich H Treasure J Carter B Schmidt U . Cytokine concentrations in people with eating disorders: A comprehensive updated systematic review and meta-analysis. Commun Med. (2025) 5:408. doi: 10.1038/s43856-025-01122-z. PMID: 41034374 PMC12488998

[11] Katar O Yildirim O . An explainable vision transformer model based white blood cells classification and localization. Diagnostics (Basel Switzerland). (2023) 13. doi: 10.3390/diagnostics13142459. PMID: 37510202 PMC10378025

[12] Cetın N Kocaturk E Tufan AK Kıraz ZK Alatas O . Immature granulocytes as biomarkers of inflammation in children with predialysis chronic kidney disease. Pediatr Nephrol. (2023) 38:219–25. doi: 10.1007/s00467-022-05530-4. PMID: 35445974

[13] Georgakopoulou VE Makrodimitri S Triantafyllou M Samara S Voutsinas PM Anastasopoulou A . Immature granulocytes: Innovative biomarker for SARS-CoV-2 infection. Mol Med Rep. (2022) 26. doi: 10.3892/mmr.2022.12733. PMID: 35551416 PMC9175277

[14] Lipiński M Rydzewska G . Immature granulocytes predict severe acute pancreatitis independently of systemic inflammatory response syndrome. Gastroenterol Rev. (2017) 2:140–4. doi: 10.5114/pg.2017.68116. PMID: 28702104 PMC5497134

[15] Maes M Hendriks D Van Gastel A Demedts P Wauters A Neels H . Effects of psychological stress on serum immunoglobulin, complement and acute phase protein concentrations in normal volunteers. Psychoneuroendocrinology. (1997) 22:397–409. doi: 10.1016/S0306-4530(97)00042-5. PMID: 9364619

[16] Nierhaus A Klatte S Linssen J Eismann NM Wichmann D Hedke J . Revisiting the white blood cell count: immature granulocytes count as a diagnostic marker to discriminate between SIRS and sepsis--a prospective, observational study. BMC Immunol. (2013) 14:8. doi: 10.1186/1471-2172-14-8. PMID: 23398965 PMC3575223

[17] Nigro KG O’Riordan M Molloy EJ Walsh MC Sandhaus LM . Performance of an automated immature granulocyte count as a predictor of neonatal sepsis. Am J Clin Pathol. (2005) 123:618–24. doi: 10.1309/73H7-K7UB-W816-PBJJ. PMID: 15743752

[18] Su Q Wang Q Cao Y . Immature granulocyte: an early predictor of strangulated adhesive small bowel obstruction. BMC Gastroenterol. (2025) 25:770. doi: 10.1186/s12876-025-04355-3. PMID: 41152714 PMC12570669

[19] Hedman A Breithaupt L Hübel C Thornton LM Tillander A Norring C . Bidirectional relationship between eating disorders and autoimmune diseases. J Child Psychol Psychiatry. (2019) 60:803–12. doi: 10.1111/jcpp.12958. PMID: 30178543

[20] Hütter G Ganepola S Hofmann W . The hematology of anorexia nervosa. Int J Eating Disord. (2009) 42:293–300. doi: 10.1002/eat.20610. PMID: 19040272

[21] Perera G Broadbent M Callard F Chang C-K Downs J Dutta R . Cohort profile of the South London and Maudsley NHS Foundation Trust Biomedical Research Centre (SLaM BRC) Case Register: current status and recent enhancement of an Electronic Mental Health Record-derived data resource. BMJ Open. (2016) 6:e008721. doi: 10.1136/bmjopen-2015-008721. PMID: 26932138 PMC4785292

[22] Macdonald AJD Elphick M . Combining routine outcomes measurement and ‘Payment by Results’: will it work and is it worth it? Br J Psychiatry. (2011) 199:178–9. doi: 10.1192/bjp.bp.110.090993. PMID: 21881095

[23] Royal College of Psychiatrists . Health of the nation outcome scales (HoNOS) (1996). Available online at: https://www.rcpsych.ac.uk/improving-care/ccqi/health-of-nation-outcome-scales (Accessed February 10, 2026).

[24] Bates D Mächler M Bolker B Walker S . Fitting linear mixed-effects models using lme4. J Stat Software. (2015) 67. doi: 10.18637/jss.v067.i01

[25] R Core Team . R foundation for statistical computing (2021). Available online at: https://www.R-project.org/ (Accessed February 10, 2026).

[26] Funayama M Koreki A Mimura Y Takata T Ogino S Kurose S . Restrictive type and infectious complications might predict nadir hematological values among individuals with anorexia nervosa during the refeeding period: a retrospective study. J Eating Disord. (2022) 10:64. doi: 10.1186/s40337-022-00586-x. PMID: 35513879 PMC9074196

[27] Keeler JL Xu C Morris R Hartland H Treasure J Himmerich H . A systematic review and meta-analysis of routine white blood cell concentrations in people with eating disorders. Neuroscience. (2025). doi: 10.1016/j.neuroscience.2025.12.045. PMID: 41423019

[28] Prabu NR Patil VP . Is immature granulocyte count a potential prognostic marker for upper gastrointestinal tract bleeding? A new road to explore. Indian J Crit Care Medicine: Peer-Reviewed Off Publ Indian Soc Crit Care Med. (2020) 24:750–2. doi: 10.5005/jp-journals-10071-23606. PMID: 33132553 PMC7584830

